# Looking into sewage: how far can metagenomics help to detect human enteric viruses?

**DOI:** 10.3389/fmicb.2023.1161674

**Published:** 2023-04-25

**Authors:** Julien Schaeffer, Marion Desdouits, Alban Besnard, Françoise S. Le Guyader

**Affiliations:** Ifremer, Laboratoire de Microbiologie, U. Microbiologie Aliment Santé et Environnement, Nantes, France

**Keywords:** sewage, human viruses, method, metagenomics, public health, molecular epidemiology

## Abstract

The impact of human sewage on environmental and food contamination constitutes an important safety issue. Indeed, human sewage reflects the microbiome of the local population, and a variety of human viruses can be detected in wastewater samples. Being able to describe the diversity of viruses present in sewage will provide information on the health of the surrounding population health and will help to prevent further transmission. Metagenomic developments, allowing the description of all the different genomes present in a sample, are very promising tools for virome analysis. However, looking for human enteric viruses with short RNA genomes which are present at low concentrations is challenging. In this study we demonstrate the benefits of performing technical replicates to improve viral identification by increasing contig length, and the set-up of quality criteria to increase confidence in results. Our approach was able to effectively identify some virus sequences and successfully describe the viral diversity. The method yielded full genomes either for norovirus, enterovirus and rotavirus, even if, for these segmented genomes, combining genes remain a difficult issue. Developing reliable viromic methods is important as wastewater sample analysis provides an important tool to prevent further virus transmission by raising alerts in case of viral outbreaks or emergence.

## Introduction

Contaminated food and water are a major pathway for the transmission of infectious diseases, this poses a global health issue, with viruses being one of the most frequently reported infectious agents. Considering the host specificity of most human enteric viruses, despite rare exception such as for hepatitis E virus, this contamination mainly occurs through contact with human sewage that have not been treated properly. People infected with enteric viruses, pathogenic or non-pathogenic, that are derived from the human gut, excreted in the stools and vomitus. Early evidence of the importance of sewage contamination with human viruses emerged in the late 1930’s, raising the question of possible transmission of poliovirus through contaminated water (Melnick, 1947; Metcalf et al., 1995). Since then, wastewater has been considered as a major source of viruses in environmental waterways. It is now acknowledged that understanding the viral composition of sewage can be informative with respect to the health of the surrounding population, as exemplified during the on-going SARS-CoV2 pandemic. Indeed, the analysis of sewage samples may provide information on the dynamics of the virus circulation at local or regional scales, measure lockdown effect and provide information on the number of infected individuals (Wurtzer et al., 2020). Using an optimized sequencing method, the spread of virus variants may also be detected in these sewage samples, making possible the early detection of shifts in emerging strains (Barbé et al., 2022).

Up to now, the major restriction of sewage analysis is that only previously described viruses are searched for and therefore detected. In 1995, a review described studies of sewage analysis up to the development of molecular biology and the possibility to detect a large range of viruses, even though many of these do not grow or grow poorly in cell culture (Metcalf et al., 1995). Since then, even if some questions on the infectivity of particles detected at the genomic level are not completely solved, many studies have demonstrated the power of molecular detection to monitor outbreaks and the usefulness of these approaches (Zheng et al., 2013; Barril et al., 2015; Miura et al., 2016).

Metagenomic developments propose a new step for the analysis of environmental samples. Next-generation sequencing (NGS) or high-throughput deep sequencing allow the massive, parallel sequencing of DNA making possible, which in theory can describe all micro-organisms present in one sample. Such NGS method has been shown to be very promising for sewage analysis as it described the diversity of virus strains circulating in the population (McCall et al., 2020; Nieuwenhuijse et al., 2020; Yang et al., 2021). In an era with increasing demographic population and an increased demand for clean water including wastewater recycling, knowing what is in our sewage will help to prevent further disease (Aarestrup et al., 2021). Consistent with this, some food such as bivalve molluscan shellfish may be contaminated by sewage, leading to consumer diseases or economic losses by food destruction (Savini et al., 2021). In these settings, human enteric viruses are frequently implicated in foodborne outbreaks (Torok et al., 2019; Savini et al., 2021). However, characterization of human enteric viruses that have short RNA genomes is challenging due to the high levels of nucleic acids from cellular organisms, the low concentrations of some viruses, and the high number of unclassified sequences (Greninger, 2018; Cobbin et al., 2021; Mazur et al., 2022). A few studies have reported the use of metagenomics to describe viral diversity in water or sewage samples, with encouraging results in the identification of different types of human virus (Kim et al., 2017; Nieuwenhuijse and Koopmans, 2017; Hendriksen et al., 2019; Martinez-Puchol et al., 2020; Adriaenssens et al., 2021). However, most of the time only amplicon-based metagenomic approaches have solved the issue of sensitivity to describe the genetic diversity of one virus genus (Suffredini et al., 2018; Fumian et al., 2019; Mabasa et al., 2022). In previous works, we found that sample pre-treatment and capture-based enrichment during the library preparation stage allowed the recovery of higher numbers of viral reads and thus the identification of longer viral contigs (Strubbia et al., 2019; Bonny et al., 2021).

Here the previously developed method was applied for the analysis of archived wastewater samples with the aim to evaluate the benefits of performing technical replicates to improve viral identification, with a focus on virus that may cause food contamination.

## Materials and methods

### Samples collection

**Figure 1:**
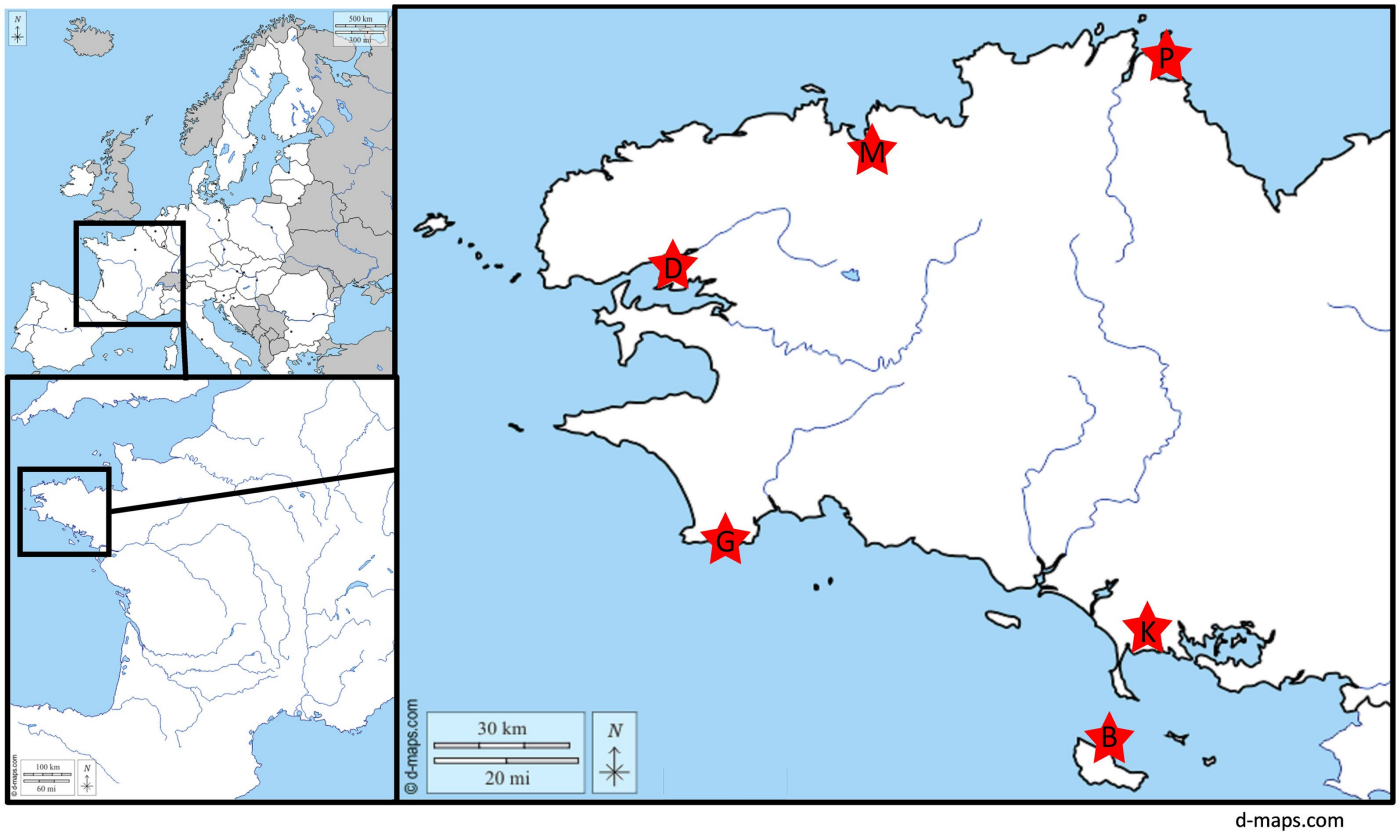
Localization of the 6 wastewater treatment plants collected for this study.

Twelve archival samples collected from six wastewater sewage treatment plants (WWTP), all located in northwestern France within 200 km of each other, were selected (Figure 1). All WWTP received sewage from a comparable number of inhabitants (8,000 to 58,000), except one which served less than two thousand inhabitants (D; Table 1). One sewage treatment plant (B) was located on a small island, 10 km away from the continent.

**Table 1:** Sample description and norovirus concentrations.

Sample	Sampling date	Inhabitants*	Average	NoV conc.**	
			Daily flow	Screening	cDNA control
2006-D	Jan. 10, 2006	1,700	496	2.45 × 10^5^	3.32 × 10^5^
2006-G	Jan. 24, 2006	26,000	889	4.34 × 10^5^	6.78 × 10^5^
2006-M	Feb. 28, 2006	58,000	6,663	2.85 × 10^5^	2.90 × 10^5^
2006-G2	Jul. 23, 2006	26,000	791	2.78 × 10^5^	2.88 × 10^5^
2007-P	Jul. 23, 2007	22,000	2,101	2.13 × 10^4^	6.51 × 10^4^
2009-P	Dec. 29, 2009	22,000	4,545	2.26 × 10^4^	5.03 × 10^4^
2012-K	Oct. 17, 2012	14,950	1,853	1.85 × 10^3^	5.52 × 10^3^
2014-K2	Feb. 05, 2014	21,500	1,876	2.02 × 10^4^	5.12 × 10^4^
2014-K1	Mar. 12, 2014	21,500	1,876	2.25 × 10^4^	6.87 × 10^4^
2014-K	Mar. 19, 2014	21,500	1,876	1.75 × 10^5^	7.21 × 10^5^
2016-B^#^	Jun. 02, 2016	8,000	1,152	2.20 × 10^5^	8.91 × 10^5^
2016-B2^#^	Jun. 06, 2016	8,000	1,152	7.16 × 10^4^	6.02×10^4^

* Treatment capacity (https://www.assainissement.developpementdurable.gouv.fr/PortailAC/data.php).

** Norovirus geometric mean concentration expressed in RNAc/L, the one-step corresponds to concentrations measured after nucleic acid extractions, and the cDNA control to concentrations measured on the cDNA synthetized for the library preparation.

# Sewage treatment plant located on a small island, 10 km away from the continent.

All samples but one (2006-D) were 24-h composite raw sewage samples, and had been stored at −20 ± 1°C in 1-liter aliquots before the metagenomics study (storage length varying from 14 to 2 years).

### Screening method

The 12 samples were concentrated from 40 ml raw sewage by adding 10 ml of 50% polyethylene glycol (PEG 6,000; Sigma-Aldrich, St-Quentin France; Sima et al., 2011). After incubation and centrifugation, the PEG pellet was suspended in 1 ml of deionized distilled water (DDW) with a vortex mixer, and nucleic acids were extracted with an automatic easyMAG extractor (bioMérieux, Lyon, France) and the NucliSENS kit (bioMérieux). Norovirus detection was performed as described below.

### Sample preparation for metagenomic analysis

**Figure 2:**
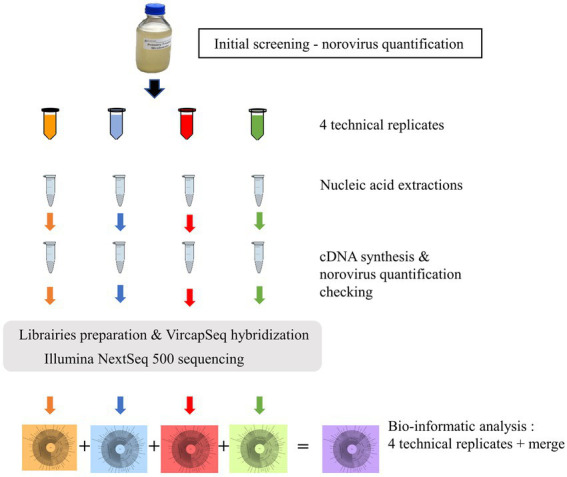
Worflow for the analysis of one sample. All 12 samples followed the same process by series of 6 for the first steps, and then by series of 8 for library preparation. A total of 49 libraries including the negative control was sequenced.

All 12 samples were analyzed under four technical replicates (Figure 2). The 48 extractions were performed by series of six samples rather than by biological replicates. After sample thawing, each of the 48 replicates (12 × 4) of 40 ml was incubated for 40 min with sodium pyrophosphate (10 mM final concentration) under gentle agitation at room temperature before three cycles of sonication for 1 min at maximum power in a cup-horn adaptor (Bandelin, HD 2200), followed by 1 min on ice (Bisseux et al., 2018; Strubbia et al., 2019). After centrifugation for 20 min at 8,000 × *g*, supernatants were recovered, the pH adjusted to 7 (using HCl), and then 10 ml of 50% of PEG 6,000 were added. After overnight incubation at 4°C under gentle agitation, the mixture was centrifuged for 1.5 h at 13,500 × *g*, the pellet was suspended in 2 ml of 0.05 M glycine buffer (pH 9).

### Purification steps and nucleic acid extraction for metagenomics analysis

The re-suspended pellets were filtrated using a cascade of 5, 1.2, and 0.45 μM filter pores (Minisart NML 17594, NML17593, PES16533) to eliminate cells and the filtrate treated with 2000 units of OmniCleave Endonuclease (Lucigen, Wisconsin, USA) for 1 h at 37°C to eliminate free DNA and RNA. Nucleic acids (NA) were extracted by adding 10 ml of a guanidium-based lysis buffer (bioMerieux) and 140 μl of paramagnetic beads (NucliSENS kit, bioMerieux; Strubbia et al., 2019). After washing steps using the eGENE-UP® apparatus (bioMérieux) following manufacturer’s instructions, extracted NA were treated with 25 U TURBO™ DNase (Ambion,Thermo Fisher Scientific, France) for 30 min at 37°C. An additional RNA purification was performed to remove DNase and PCR inhibitors (RNA Clean & Concentrator™ -5 kit, Zymo Research, Irvine, USA).

### Norovirus quantification

Norovirus quantification was performed for the two main genogroups I and II (GI and GII) using primers and probes targeting the conserved region at the beginning of ORF2 (Kageyama et al., 2003; Loisy et al., 2005; da Silva et al., 2007; Svraka et al., 2007). The *r*RT-PCR was carried out with the Ultrasense One-step quantitative RT-PCR system (Life Technologies, France) using 5 μl of extracted NA per well (final volume of 25 μl) in duplicate on a Mx3000P QPCR System (Agilent Technologies, France). Standard curves based on double strand DNA (gBlocks, IDT USA) corresponding to nucleotides 4,484 to 5,668 of the GI.1 Norwalk virus (Genbank M87661) or nucleotides 4,217 to 5,355 of the GII.4 Houston virus (Genbank EU310927), were included in each run. Concentrations range from 8.1 and 3.4 RNA copies/μl to 8.1 × 10^4^ and 3.4 × 10^4^ RNA copies/μl for norovirus GI.1 and GII.4, respectively.

Two negative amplification controls (sterile, RNase-free water) were included in each amplification series and filter tips and dedicated rooms were used to prevent false-positives. Standard curves included in each run were analyzed and retained for NA quantification if amplification efficiencies were comprised between 85 and 110%. For the screening, concentrations were calculated per sample by adding GI concentrations to GII. When samples were prepared for metagenomics, concentrations obtained for each replicate (4 per sample), were calculated separately, then GI and GII concentrations were added and the four values obtained were used to calculate the geometric mean concentration (GMC) per sample.

### Library preparation and sequencing

Libraries were prepared by series of eight. All the 48 NA extracts were transcribed into cDNA using the Superscript II (Invitrogen, Saint-Aubin, France) and random hexamers (New England Biolabs (NEB), USA). Completion of this step was checked by PCR quantification for norovirus GI and GII as described above, except that enzyme mix was replaced by Platinum taq (ThermoFisher Scientific) and the RT step was removed from the thermal profile.

After double strand DNA synthesis using second strand reaction buffer from NEBNext Ultra RNA Library prep, physical fragmentation of the DNA was performed using sonication (Ultrasonicator Covaris M220, duty factor 5%, peak power: 75, cycles per dust: 200 for 195 s, Woburn, MA) and libraries were prepared using the kit KAPA Hyper Prep (Roche), according to the manufacturer’s instructions. Viral sequences were enriched using VirCapSeq-Vert probe panel (Roche; Briese et al., 2015; Strubbia et al., 2020). A negative control, in the form of sterile RNase-free water, was treated in parallel from cDNA synthesis onwards. All 49 libraries were sequenced using Illumina NextSeq 500 technologies to generate 2×150 bp reads.

### Bioinformatic analysis

Bioinformatic analysis was performed using a Nextflow pipeline as previously described (Bonny et al., 2021). For the first part of the analysis, reads from each library as well as reads merged from all four libraries per sample were considered (5 analysis for each sample; Figure 2). Briefly, Fastq were trimmed using fastp with a quality threshold at 25. Clean reads were deduplicated (CD-hit) and mapped to remove bacterial RNA reads (Silva RNA database) and PCR duplicates. *De novo* assembly was then performed using metaSPAdes with kmer length 21, 33, 55, 77, 99 (Nurk et al., 2017). Contigs longer than 500 bp were filtered and identified using BLASTn compared to *nt* database (download 2022-05-08) with an e-value of 10^−5^, and *nr* database (download 2021-02-06) using diamond with an evalue of 10^−3^. When both approaches gave results, the BLASTn match was kept. To evaluate the coverage of each contig, post-process reads were mapped using Bowtie2 (v2.3.0; Langmead and Salzberg, 2012) on the metaSPAdes contigs. Multi-mapped reads were removed as a source of potential overestimation of the abundance. Coverage was calculated using the Lander-Waterman equation $C=\frac{N\times \left(R\right)}{L}$ , where *C* is the coverage, *N* is the number of reads, *R* the length of the reads (150 bases) and *L* the length of the considered contig (Lander and Waterman, 1988). After this step, only the “merged” dataset was considered for each sample.

Taxonomic identification was done using Entrez direct tool, the taxid allowed to extract information at a defined taxonomic level. Reads per millions (rpm) were calculated using the number of reads per family and the total number of reads after trimming and deduplication. The heatmap was done using Graphpad prism v 9.0.0 (GraphPad Software, sanDiego, CA, US).

Genotyping of viral sequences was performed using several web-based tools, and only contigs represented more than 100 reads were considered. For norovirus, sapovirus or hepatitis A & E virus sequences, the identification was done using the respective online Typing Tools and for rotavirus the Rotac tool (Maes et al., 2009; Kroneman et al., 2011). Other contigs identified as enterovirus, mamastrovirus and picobirnavirus sequences were characterized using Genome Detective (Vilsker et al., 2019).

Phylogenetic analysis were performed by aligned sequences using Muscle or ClustalW2, followed by IQ-TREE algorithm to choose the best fit model according to the BIC (Bayesian Information Criterion) and to construct phylogenetic trees with a boostrap of 1,000.

## Results

Samples were collected from sewage treatment plants from relatively small communities over a period of 10 years (Table 1). Eight samples were collected during winter months (October to March) and four during summer (June and July). Despite these different seasons, following the initial screening, all samples were positive for norovirus GI and/or GII and there were no differences in total norovirus concentration between winter and summer samples (Table 1).

For metagenomics, the 12 samples were treated and extracted 4 times each (Figure 2).

After the nucleic acid extractions of the four replicates from each of the 12 samples, 29 were positive for norovirus GI and 40 for norovirus GII. After cDNA synthesis, 22 extracts were positives for norovirus GI and 45 for norovirus GII (data not shown). One replicate of sample 2009-P did not give a GI or GII Ct value and none of the reads were identified as norovirus. Norovirus concentrations, expressed as GI + GII, obtained after cDNA synthesis were similar or half-a log higher compared to concentrations obtained for the RNA (Table 1). All four replicates were kept for the library preparation to ensure that at least three replicates would be obtained.

Among the 818 million of reads obtained after the Illumina sequencing, 44 libraries gave comparable read numbers ranging from 1,565,594 to 85,453,164 reads, and four libraries yielded a low number of reads (17,134 to 736,618 reads; Table 2). More than 35,000 reads were obtained from the negative control but after the different cleaning steps and identification steps no viral read was identified.

**Table 2:** Overview of read numbers obtained for the 48 librairies and the merge value for the 12 samples.

Name	Rep.*	Raw reads	Clean reads	Identified reads	Viral reads	Coef. of var. %**	Viral reads %***
2006-D	1 2 3 4	4,506,340 2,402,894 3,606,002 3,206,050	688,804 393,000 393,464 312,984	387,983 266,782 289,073 120,321	361,518 249,453 288,018 119,597	40	
	Merge	13,721,286	1,788,252	1,323,336	1,272,409		71
2006-G	1 2 3 4	9,541,300 11,217,902 2,966,010 6,423,356	2,260,746 1,530,728 356,374 598,924	1,488,848 1,274,720 209,670 340,585	990,656 1,261,356 208,750 335,904	88	
	Merge	30,148,568	4,743,756	3,699,942	3,167,343		67
2006-M	1 2 3 4	17,134 47,644,656 14,262,232 76,825,830	5,236 4,573,184 1,191,024 7,576,116	2,582 4,187,147 930,015 7,053,620	2,582 4,173,108 928,085 7,026,021	106	
	Merge	138,749,852	13,343,538	12,436,823	12,386,553		93
2006-G2	1 2 3 4	29,093,288 8,137,376 85,435,164 1,565,594	4,081,130 680,206 8,064,160 132,970	3,522,608 562,770 7,529,846 16,431	3,041,567 562,451 7,509,918 14,371	123	
	Merge	124,231,422	12,953,180	11,718,516	11,211,954		87
2007-P	1 2 3 4	5,774,206 9,458,542 8,181,372 3,455,326	92,5,076 919,580 862,832 338,988	464,453 620,486 676,385 162,431	410,478 611,586 673,825 158,685	50	
	Merge	26,869,446	3,044,114	2,149,086	2,061,661		68
2009-P	1 2 3 4	3,772,094 4,836,042 372,442 5,450,408	698,092 587,498 37,518 448,972	374,629 407,026 22,264 209,349	213,068 300,583 17,321 180,879	67	
	Merge	14,430,986	1,770,770	1,107,117	795,808		45
2012-K	1 2 3 4	14,176,218 9,356,070 3,554,214 36,261,558	4,587,968 2,572,568 450,022 2,545,060	1,060,613 2,110,438 197,616 2,086,422	558,029 1,684,264 137,633 2,038,462	82	
	Merge	63,348,060	10,150,928	5,871,134	4,810,896		47
2014-K2	1 2 3 4	1,856,764 3,872,212 52,270,136 736,618	558,526 588,342 3,846,026 263,830	256,651 324,504 3,371,485 20,151	98,446 213,169 3,300,670 2,553	109	
	Merge	58,735,730	5,256,534	4,041,606	3,686,030		70
2014-K1	1 2 3 4	12,176,666 16,887,440 3,298,316 3,114,878	2,336,528 1,544,840 298,646 239,354	1,514,188 1,115,033 162,226 128,259	984,878 779,709 150,098 118,964	87	
	Merge	35,477,300	4,418,862	3,153,740	2,248,319		51
2014-K	1 2 3 4	9,137,872 12,253,584 5,421,844 39,869,020	1,418,210 1,132,042 447,440 4,487,688	877,541 858,364 222,963 3,574,163	789,752 850,963 218,749 3,444,980	177	
	Merge	66,682,320	7,485,152	5,957,168	5,720,168		76
2016-B	1 2 3 4	10,936,716 68,870,930 39,108,178 21,645,010	2,801,174 8,652,778 3,875,918 2,476,722	1,762,383 7,858,150 3,124,556 2,031,273	1,440,351 7,735,805 3,042,426 2,012,830	81	
	Merge	140,560,834	17,795,292	15,290,037	14,708,048		83
2016-B2	1 2 3 4	24,749,188 48,291,662 715,534 31,200,266	5,210,512 13,195,394 104,526 3,267,994	3,080,758 11,603,668 76,346 2,112,003	2,350,567 9,160,827 75,782 2,029,485	117	
	Merge	104,956,650	21,772,204	17,266,476	14,007,068		64
Control		35,160	11,458	414	0		

* Data are provided for each replicate (rep.) and for the merge value of all four replicates for each sample (grey line),

** Coefficient of variation is calculated as the ratio of the standard deviation σ to the mean μ , $c_{v}=\frac{\sigma }{\mu .}$

*** Percentage of viral reads calculated based on viral reads divided by clean reads, and multiplied by 100.

**Figure 3:**
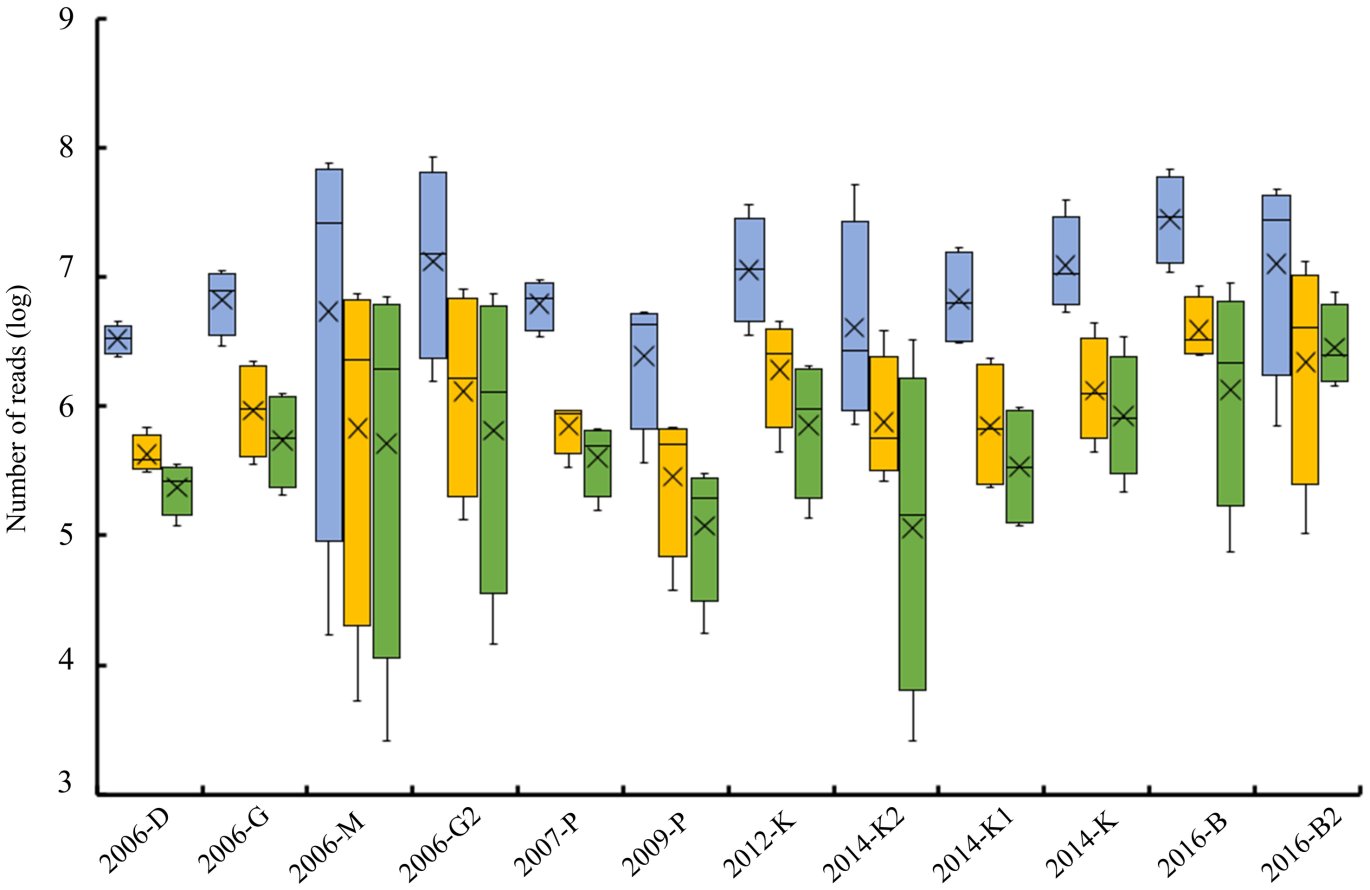
Schematic representation of reads obtained for the four replicates analyzed per sample. Boxplots represent the number of raw reads (blue box), after trimming and filtering (orange box), and viral reads (green box) for each sample. Bloxplots show the minimum, 25th percentile, average (cross), median (line), 75th percentile and maximum number of reads.

Some samples (2006-M, 2006-G2, 2014-K2, and 2016-B2) presented a large variability between replicates when considering raw reads, clean reads or viral reads, while some others (2006-D, 2006-G, 2007-P, and 2014-K) presented a similar number of reads for all replicates (Figure 3). The coefficient of variation calculated to evaluate viral reads variability between replicates showed over 100% for five samples, and only two samples displayed a coefficient of variation ≤50% (Table 2). In addition to this between analysis replicate, data from the 4 replicates were merged and analyzed together for each sample. When considering merge analysis, the variability between samples in terms of raw reads decreased (from 13,721,286 reads obtained for sample 2006-D to 140,560,834 reads for sample 2016-B) compared to individual libraries (from 17,134 reads obtained for replicate 1 sample 2006-M to 85,435,164 reads for replicate 3 sample 2006-G2; Table 2). The percentage of viral reads varied from 45% (sample 2009-P) to 93% (sample 2006-M) after merged replicates analysis (Table 2).

**Figure 4:**
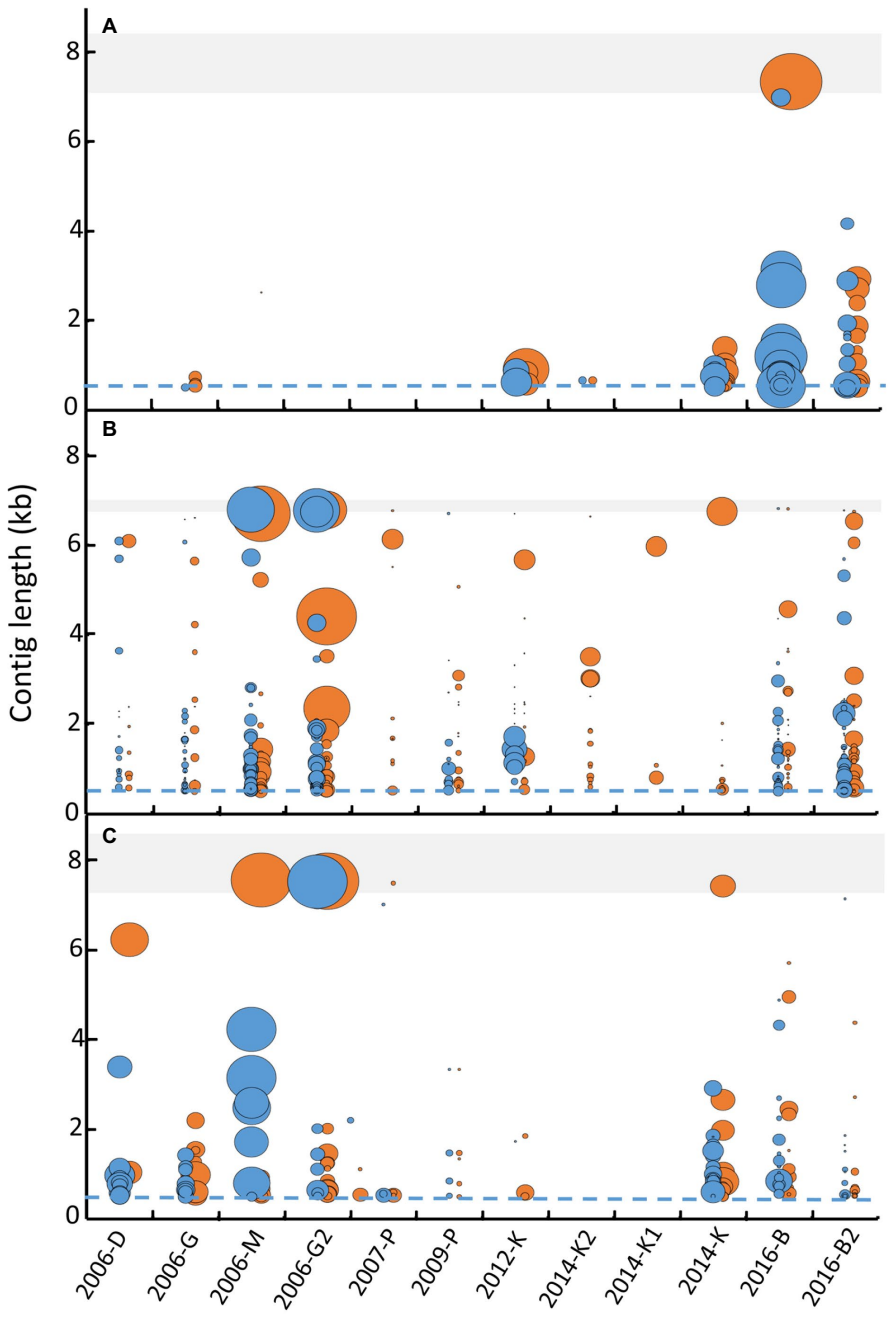
Comparison of contig lengths identified after separate librairy analysis or merged analysis for three of ssRNA virus genus. Contigs longer than 500 bases (dashed blues lines) obtained for each library (blue dots) and the merge analysis (orange dots) were reported for the 12 samples for enterovirus **(A)**, mamastrovirus **(B)**, and norovirus **(C)**. For each viral viral family, the shaded area represents the expected size of complete genome (7.2 to 8.5 kb for enterovirus, 6.8 to 7 kb for mamastrovirus and 7.3 to 8.3 kb for norovirus). The size of the dots represents the coverage obtained for each contig (calculated by the number of bases obtained from all reads divided by the length of the contig) and represented as a ratio between coverage of the contig considered divided by the highest coverage obtained for the genus.

To further decide which approach allows better viral identification, the length of contigs obtained for three genera of enteric viruses (mamastrovirus, enterovirus and norovirus), were compared considering contigs obtained for each separate replicate (blue) and with merged replicates (orange; Figure 4). For mamastrovirus, only samples 2006-M and 2012-K presented a longer contig with one replicate analysis compared to the merged replicate, however these long contigs had a low coverage (Figure 4B). For all the other samples, the merged-replicates analysis provided longer contigs with increased coverages, for example for samples 2007-P, 2012-K, 2014-K1, 2014-K, and 2016-B2. For enterovirus, the merged-replicates analysis increased the contig length for all samples except two. A clear improvement was observed for sample 2016-B for which the merged analysis allowed the assembly of a complete genome that remained fragmented in the separate replicates, and for sample 2016-B2 by increasing the coverage of the different contigs (Figure 4A). Similar observations were obtained for norovirus, with complete genomes identified using the merged-replicates analysis and a good coverage as observed for sample 2006-M (Figure 4C). The long contig obtained with only one replicate for sample 2016-B2 was based on few reads and was not confirmed with the merged-replicate analysis.

Therefore, only the merged-replicate datasets were used for subsequent analyses.

### Viral family identification

**Figure 5:**
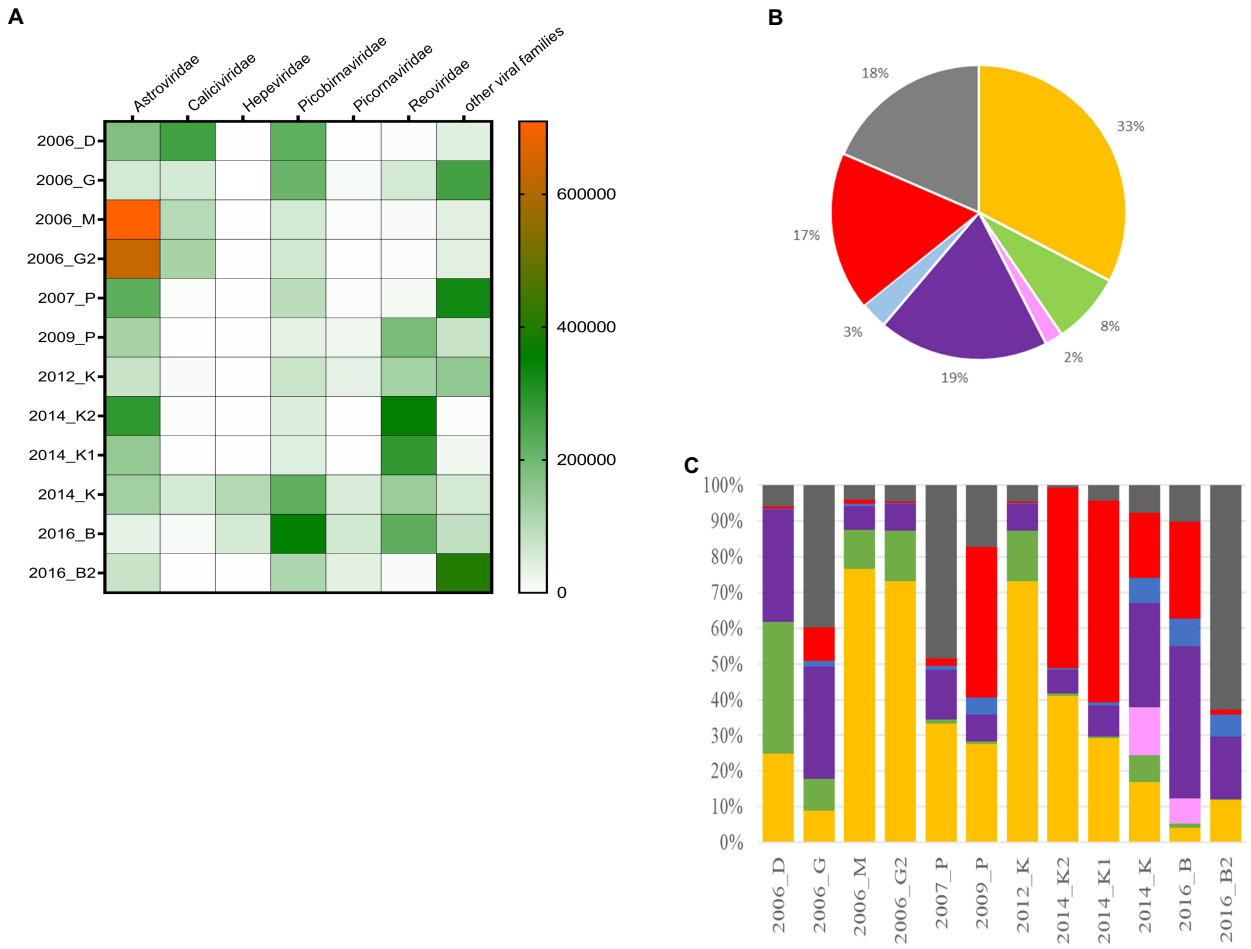
Distribution of the different viral families detected in the 12 samples. **(A)** Heat-map representing the abundance of the six selected families and the other viral read abundance all included as ‘other families’, the scale bar expressed the number of rpm. **(B)** Distribution of cumulated reads obtained for all samples expressed in % for the different families. **(C)** Relative abundance of the different families identified for each sample. *Astroviridae* (orange), *Caliciviridae* (green), *Hepeviridae* (pink), *Picobirnaviridae* (purple), *Picornaviridae* (blue), *Reoviridae* (red), and other families (dark grey).

An initial taxonomic identification was conducted directly on the clean reads of merged replicates. Among the 76,076,257 viral reads, the large majority of reads were identified as sequences related to viral families infecting humans such as *Astroviridae, Caliciviridae, Hepeviridae, Picobirnaviridae, Picornaviridae*, and *Reoviridae* (Figure 5B). Reads belonging to the *Astroviridae* and *Picobirnaviridae* families were detected in all samples, representing 51% of total reads (Figures 5A,B). A large proportion of reads (17%) were identified as belonging to the *Reoviridae* family, but these were not detected in all samples. The same observation can be made for *Caliviridae* or *Picornaviridae* reads. For eight samples, viral reads belonging to the six families cited above represented the majority of reads with less than 10% of reads identified as other viruses. Conversely, for the four other samples (2006-G, 2007-P, 2009-P, and 2016-B2), the proportions of ‘other viruses’ (in which we included different families such as unclassified ribovirus, *Nodaviridae*, *Tombusviridae*, *Virgaviridae*, or *Marmaviridae*) ranged from 17 to 63% (Figure 5C). Reads belonging to the *Hepeviridae* family were identified only in two samples (2014-K and 2016-B).

**Figure 6:**
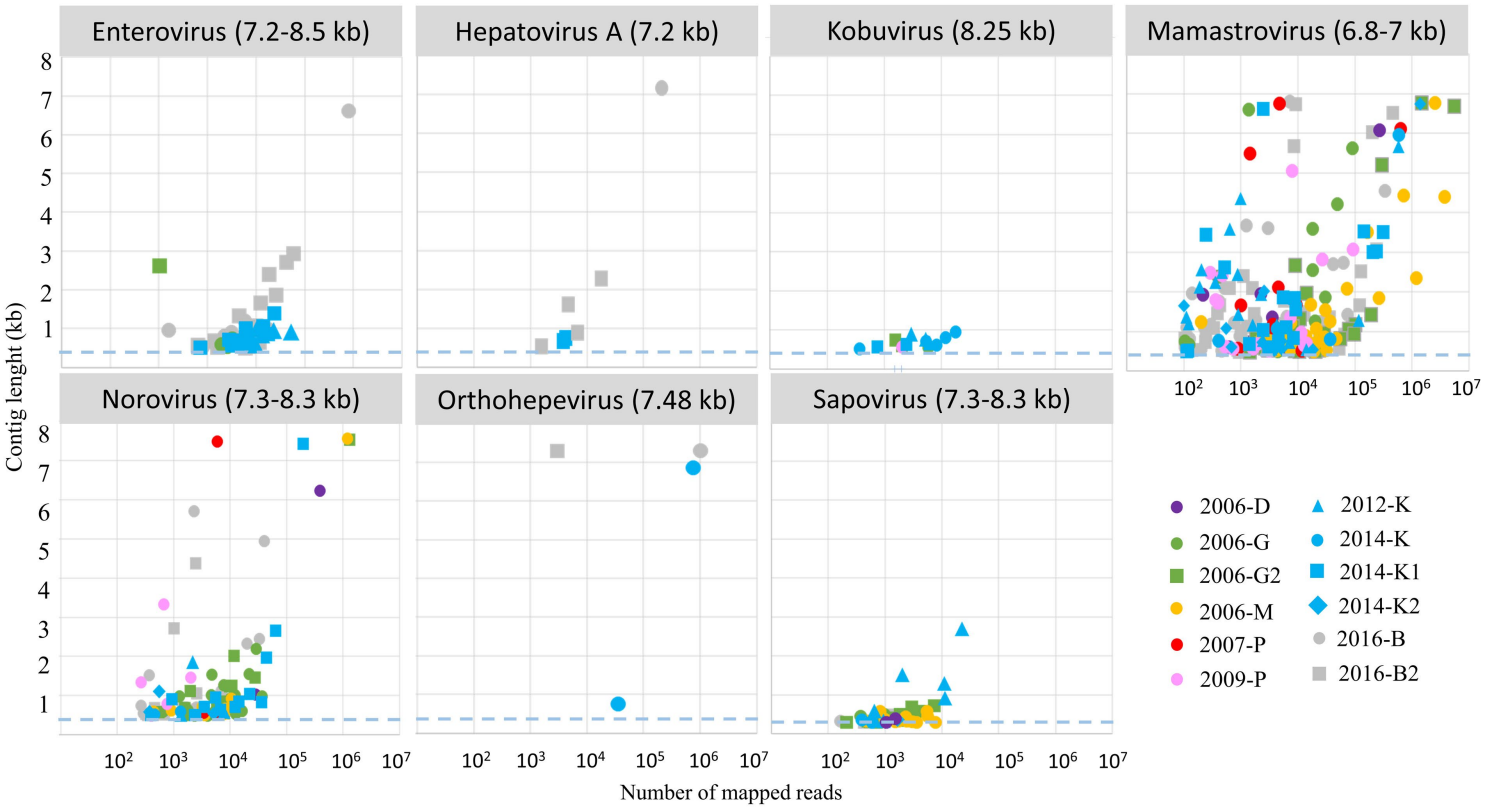
Relationship between number of mapped reads and associated length contigs for 7 ssRNA genus selected for the study. Only clusters with more than 100 reads were considered for contig assembly, the dashed blue lines represented the threshold selected for contig identifications (0.5 kb), the expected size of complete genome is written into brackets after the virus name. Each WWTP is represented by one color, and each sample by a symbol.

After assembly, contigs were identified using different tools (see method section) and 1,375 contigs were identified as belonging to nine selected viral families comprising known human pathogens (Figure 6): seven ssRNA virus families and two dsRNA viruses with segmented genomes (rotavirus and picobirnavirus). For more precise identification, we focused on contigs longer than 1,000 bases, except for the segmented viruses such as rotavirus (sequences representing at least 97% compared to reference segment length) or picobirnavirus (sequences representing at least 90% compared to reference segment length). For mamastrovirus, considering the high number of long contigs, only complete genomes were analyzed thoroughly (Supplemental Table S1). For picobirnavirus, numerous contigs were obtained with a high number of complete segments (1 and 2), mainly related to human or dog strains.

Long contigs or complete genomes were obtained for different viral genera with a clear relationship to read numbers (Figure 6). However, for nororovirus or orthohepevirus, even with less than 10^4^ reads, complete genomes were assembled. For kobuvirus, all the different samples provided a low number of reads and contigs obtained were all under 1 kb.

Within the *Caliciviridae* family, when considering contigs longer than 1 kb obtained with more than 100 reads, 32 contigs were identified as norovirus and three as sapovirus. A large range of norovirus read numbers per sample was observed from 264 reads (sample 2009-P) to 1,309,920 reads (2006-G2; Figure 6; Supplemental Table S1). Only sample 2012-K presented both norovirus contigs and sapovirus contigs. In this sample, two sapovirus contigs were identified as a GII (ON807341, ON807342), and the third one as a GII.1 (ON807343). All the other contigs were identified as norovirus, with sequences identified either in the ORF1 or ORF2 typing regions (Table 3).

**Table 3:** Heatmap profile showing the relative abundance the different norovirus genotype sequences detected in the 12 samples.

	ORF1									ORF2									
	GI.[P1]	GI.[P8]	GI.[P9]	GI.[P13]	GII.[P7]	GII.[P17]	GII.[P21]	GII.[P25]	GII.[P31]	GI.1	GI.3	GI.8	GI.9	GII.3	GII.6	GII.7	GII.12	GII.14	GII.17
2006-D	0	0	0	0	230,961	0	0	0	0	0	0	0	0	0	215,421	0	0	0	0
2006-G	0	0	1,257	1,104	19,173	0	315	0	0	0	3,046	0	351	778	13,198	1,332	1,098	4,246	0
2006-M*	67	0	0	0	91,892	0	0	0	0	0	32	0	0	0	89,428	580	0	1	0
2006-G2*	1,019	0	2,249	0	104,416	0	0	0	0	560	0	0	1,017	991	101,242	1,179	0	537	0
2007-P*	0	0	0	0	3,020	0	0	0	0	0	0	0	0	0	1,918	4	0	1,829	0
2009-P	0	0	0	0	2,067	0	0	0	0	0	0	0	0	0	614	0	0	0	0
2012-K	0	0	0	0	229	0	0	0	0	0	0	132	0	0	230	0	0	770	0
2014-K2	0	0	0	0	1,054	0	0	0	0	0	0	0	0	0	104	0	0	0	0
2014-K1	0	103	0	0	0	3	0	0	0	0	0	0	296	0	0	0	0	0	3
2014-K*	0	0	0	0	50,292	325	0	2,805	311	0	0	0	0	0	34,240	0	0	0	2,361
2016-B	1	0	1	0	573	1,482	0	2,262	0	0	0	0	1	0	597	2	0	16	1,823
2016-B2	16	0	0	0	199	166	0	18	0	0	182	0	1	1	46	4	0	0	67

The number of reads per million (rpm) showing a positive blast with the different genotypes identified is reported for each sample, for the two main ORF allowing the typing. The stars indicate samples in which a complete genome was identified. Green case: no rpm, yellow case < 20,000 rpm, red case: >20,000 rpm.

No clear relationship between norovirus concentrations measured by RT-qPCR, and the abundance of norovirus reads, was observed. For example, sample 2012-K that presented the lowest norovirus concentration and 1,360 rpm, whereas samples 2014-K1 or 2016-B2 provided lower rpm numbers with norovirus concentrations 10 times higher. Two samples (2006-D and 2007-P) for which none of the cDNA replicates were positive for norovirus GI by PCR provided no norovirus GI reads, while some GII reads were identified. Similarly, no norovirus reads were obtained from the replicate of sample 2009-P that did not provided any Ct value. Norovirus sequences were genotyped using the Typing tool and the Noronet database, for the ORF1 and ORF2 regions separately (Table 3). Overall, we identified four different GI P-types, five GII P-types, four GI genotypes and six GII genotypes. Four complete genomes assigned as GII.6[P7] (ON706286, ON706287, ON706288, and ON706289) were identified in four samples collected in 2006, 2007 and 2014. Two samples collected in 2006 and 2016 provided almost complete genomes of two GII.6[P7] strains (ON706290 and ON706291). For some samples, despite a high number of reads, the contigs obtained did not allow a more precise identification than the genogroup assignation, because of the contig position on the genome outside of the typing regions in ORF1 or 2. Samples collected serially in the same WWTP show a high variability in the identified circulating norovirus strains. For instance, in 2014 in WWTP K, most of contigs were identified as GII.6 and GII.[P7], which were not detected in the sample 2014-K1 collected one week before. However, this sample provided very low number of reads.

**Figure 7:**
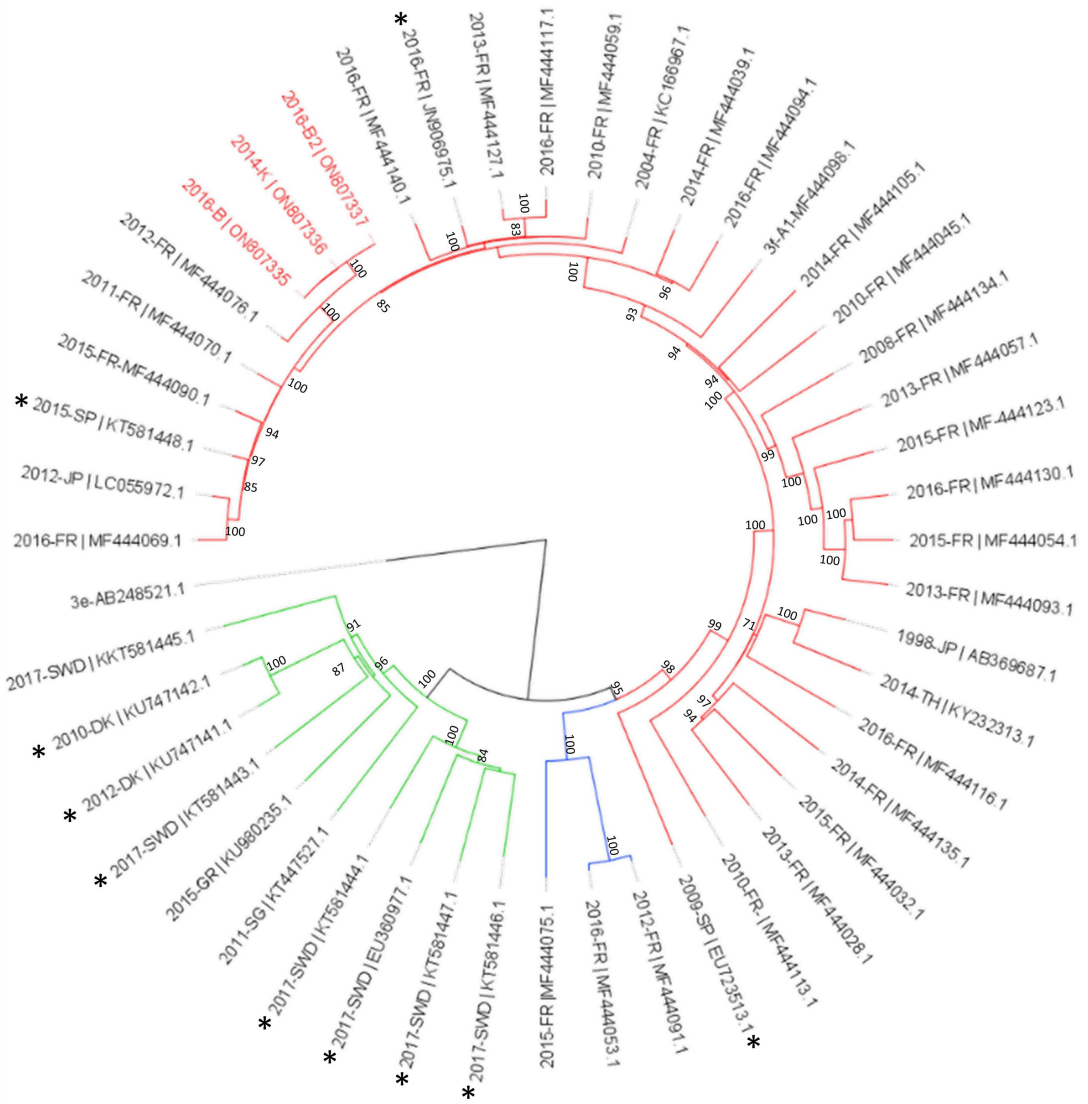
Phylogenetic analysis of HEV-3 subtype f subclusters using full genomes sequences identified in this study. The three complete genomes detected in this study (written in red) were aligned with reference sequences of subtype HEV-3f-A1 (red lines), HEV-3f-A2 (blue lines) and HEV-3f-B (green lines; Munoz-Chimeno et al., 2022). Sequences of animal origin are identified with an asterisk. The tree was constructed using IQtree with the GTR + F + I + G4 substitution model with ultrafast bootstrap (bootstrap = 1,000) analysis, boostrap values greater than 70% are shown. Rooted tree was drawn using FigTree.

Among the *Hepeviridae* family, four contigs were identified as orthohepevirus from samples collected in site B and K (Figure 6). One contig was short (<1 kb), but the three other contigs were complete genomes (ON807340, ON807339, ON807338) identified as hepatitis E virus, genotype 3f (blast identification 98%, Supplemental Table S1). Sample 2016-B2 gave a complete genome (7,254 bases) assembled from a relatively low number of reads (2,991 reads), corresponding to a mean coverage of 123 reads. The two other complete genomes were obtained with much higher numbers of reads (737,250 reads for sample 2014-K and 1,012,569 reads for sample 2016-K) with mean coverages of 32,162 and 41,573 reads, respectively. These three contigs were identical (Figure 7) and similar to strains circulating in France 2011 and 2012.

Among the *Picornaviridae* family, 40 contigs were identified. Of these contigs, 16 were assigned as Beihai or Whenzhou picorna-like viruses, nine as salivirus A, two as parechovirus A, two as hunnivirus, one as palmarnavirus and the other contigs remained unsassigned. A full genome of hepatitis A virus was obtained from a sample collected in WWTP B, the only site in which hepatitis A virus contigs longer than 1 kb were obtained. The two other contigs obtained from this sampling site B were identical to the full genome, identified as a genotype IA (Supplemental Table S1).

This WWTP B showed also the highest diversity for enterovirus sequences (Table 4). Ten enterovirus contigs were detected in seven samples collected in three WWTP (G, K and B), and were most frequently identified as enterovirus type A and B. One complete genome (6,619 nucleotides) of a coxsackievirus A9 strain was identified in the first sample collected in June in WWTP B. A week later in the same WWTP, up to 5 different contigs were identified, including a coxsackievirus A9 sequence presenting 65% homology over 647 bases with the previous one. In two samples collected in 2016 in WWTP B, two contigs were identified as the enterovirus type EV-A114, an enterovirus that was described in childrens stool samples in India (Deshpande et al., 2016).

**Table 4:** Heatmap profile showing the relative abundance for enterovirus sequences detected in the 12 samples.

Sample	Enterovirus A		Enterovirus B			Enterovirus C		Enterovirus J
	Untyped	A114	Untyped	E-9	CV-A9	Untyped	CV-A22	Untyped
2006-D	0	0	0	0	0	0	0	0
2006-G	0	0	0	0	0	1,085	0	526
2006-M	0	0	0	0	0	0	0	0
2006-G2	0	0	0	0	8	0	0	0
2007-P	0	0	0	0	0	0	0	0
2009-P	0	0	0	0	0	0	0	0
2012-K	0	0	11,124	0	0	0	0	0
2014-K2	0	0	211	0	0	0	0	0
2014-K1	0	0	0	0	0	0	0	0
2014-K	0	0	9,388	0	0	1,693	653	557
2016-B	943	1,436	174	0	43,685	53	0	0
2016-B2	2,912	1,291	4,528	528	0	102	0	0

The number of reads per million (rpm) showing a positive blast with the different strains identified is reported for each sample. Green case: no rpm, yellow case < 20,000 rpm, red case: >20,000 rpm.

For the *Astroviridae* family, 128 of contigs longer than 1 kb were assigned as belonging to mamasatrovirus genus. A large majority, 63% (80 contigs) of the contigs, were identified as mamastrovirus 1 (HAstV-1), using the genome detective web tool. This genus was represented in all samples. Feline astrovirus 2 (FAstV-2) was the second most identified genus (17%) with 22 contigs. Other contigs were identified as mamastrovirus 2 (HAstV-2) 1 contig, mamastrovirus 5 (HAstV5) 1 contig and mamastrovirus 9 (HAstV-9) 3 contigs. Twenty one contigs were close to reference sequences that were not been approved as a species by the ICTV, but belong to the mamastrovirus genus: 16 contigs of Human astrovirus MLB2 (HAstV-MLB2), 4 contigs of the Culex Bastrovirus-like virus and 1 contig of Canine astrovirus (CaAstV-1). Twelve long contigs (more than 6 kb) were found in all samples except sample 2009-P.

**Figure 8:**
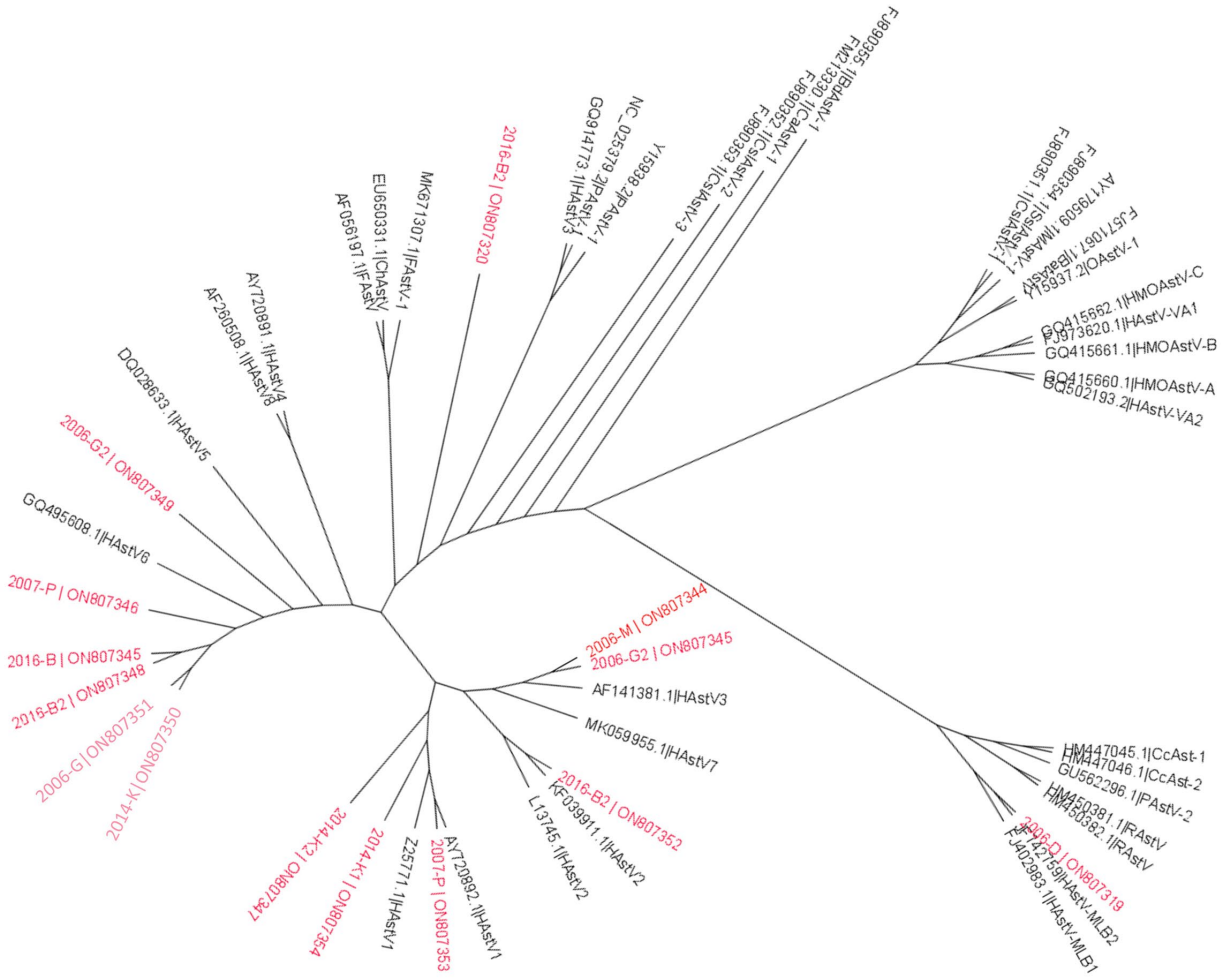
Phylogenetic analysis of *Astroviridae* family based on RdRp protein. Reference sequences were selected from the main genus of the *Astroviridae* family, sequences identified in this study are written in red. The tree was constructed using IQtree with the LG + F + R5 substitution model with ultrafast bootstrap analysis. None-rooted tree was drawn using FigTree.

Among the complete genomes obtained in the different samples, six clustered to HAstV-6, and six to HAstV-1. Phylogenetic analysis of the complete RdRP protein sequence confirmed identification of the complete HAstV genomes compared to previously available sequences in GenBank (Figure 8).

*Picobirnaviridae* reads were identified in all samples (Figure 5A), and up to 793 contigs (longer than 500 bases) were identified as picobirnavirus. The number of contigs varied largely depending on the samples, from 6 contigs in sample 2014-K2 to 259 contigs in sample 2016-B2. Picobirnavirus genome is constituted of two segments and 13% of contigs matched to segment 1 (encoding hypothetical proteins) with four sequences being almost complete (around 2,525 bases). All the other contigs (87%) were identified as segment 2 (RNA-dependant RNA polymerase coding gene), with 70 contigs being complete sequences (1745 bases). Among these complete or nearly complete segments, only 11 showed sequence similarity with human picobirnavirus (NC_007027.1). The other contigs showed sequence similarity with otarine picobirnavirus (NC_034160.1; 46 contigs), porcine picobirnavirus (NC_029802.1; 12 contigs), roe deer Picobirnavirus (NC_040753.1; 4 contigs) and 1 Chicken picobirnavirus (NC_040439.1; 1 contig).

Rotavirus genes were detected in all samples with 26 complete genes segments being sequenced in several samples (Table 5). Only one complete VP1 gene was obtained, as well as one VP7, while five, VP2, eight VP3, two VP4, two VP6 were obtained. Regarding non-structural proteins, only two NSP3 genes, one NSP1 and one NSP2 were identified as full sequences. Twenty-seven nearly complete genes were identified. Contigs coding for VP7 and VP4 allowed the identification of RVA G1P[8], for which most of the other genomics segments were identified in all samples (Table 5). Moreover, in samples 2016-B and 2014-K1, all genomics segments previously identified as belonging to this strain could be sequenced, likely representing full RVA G1P[8] Wa-like genomes (Table 5). Evidence for co-circulating RVA strains were observed in several samples. For example, four samples displayed three different complete VP7 gene segments (G1, G3 and G9), while 6 other samples contained either G1 and G3 or G1 and G9 complete VP7 gene segments. In most samples, the VP4 genes were identified as P[8] except for sample 2016-B2 for which a P[4] and a P[1] VP4 genes were also identified. In other samples, two different VP1 sequences, or up to 3 VP2 or NSP3, could be detected. Segments that may correspond to the constellation of the Human DS-1 like strain were detected in three samples while segments from the AU-1-like constellation were identified in 9 samples (Table 5, red and orange, respectively). NSP4 (only one complete gene) and NSP5 segments were rarely detected, and the few sequences obtained were identified as belonging to the constellation of Human Wa-like strain. A NSP3 sequence identified as T6 was identified once (Table 5, purple).

**Table 5:** Genotype constellation of human RVA sequences identified.

Sample	VP7 (1062)	VP4 (2362)	VP6 (1356)	VP1 (3302)	VP2 (2687)	VP3 (2592)	NSP1 (1581)	NSP2 (1059)	NSP3 (1074)	NSP4 (751)	NSP5 (666)
2006-D	G3	P[8]	I1	R1	C1	M1		N1	T1	E1	H1
2006-G	G1	P[8]	I1	R1	C1	M1	A1	N1	T1		H1
	G3										
2006-M	G1	P[8]	I1	R1	C1	M1			T1		
	G3										
2006-G2	G1	P[8]	I1	R1	C1	M1			T1		H1
	G9										
	G3										
2007-P				R1	C1	M1	A1		T1		
2009-P	G1	P[8]	I1	R1	C1	M1		N1	T1		
	G9								T2		
2012-K	G1	P[8]	I1	R1	C1	M1	A1	N1	T1		
	G9			R2					T2		
	G3										
2014-K2	G1	P[8]	I1	R1	C1	M1	A1	N1	T1	E1	
	G3										
2014-K1	G1	P[8]	I1	R1	C1	M1	A1	N1	T1	E1	H1
	G9										
2014-K	G1	P[8]	I1	R1	C1	M1	A1	N1	T1	E1	
	G3										
2016-B	G1	P[8]	I1	R1	C1	M1	A1	N1	T1	E1	H1
	G9			R2	C2	M2		N2	T2		
	G3						A3		T6		
2016-B2	G1	P[8]	I1	R1	C1	M1	A1	N1	T1		H1
	G9	P[4]	I2	R2	C2				T2		
	G3	P[1]			C3				T3		
% identity	92.8–98.9	94.5–98.9	97.7–98.9	97.2–98.2	88.6–98.6	91.2–99.1	97.6–98.6	98.1–98.5	96.2–98.7	98.46–98.80	

Sequences identified for the different genes are reported for each sample. The Wa-like genotype is shown in green, DS-1-like in red, AU-1-like in orange, and P [6] in purple, following genotype constellations (Matthijnssens and Van Ranst, 2012), sequence identities are expressed in % for the lowest and highest homologies found. The number written after each segment is the expected length of the gene.

## Discussion

As mentioned above, the development of metagenomic approaches opens new perspectives in environmental virology through their capacity to sequence all the genomes present in a sample. Strong evidence of the potential of metagenomics in environmental virology have been provided for example by the TARA expedition which brought new data on the ocean viromes or by the analysis of sewage viromes (Nieuwenhuijse et al., 2020; Sunagawa et al., 2020). Using metagenomic analysis, a study was able to suggest a conceptual model of virus circulation considering human and livestock inputs throughout the fresh-marine continuum of a river catchment (Adriaenssens et al., 2021). A good complement to such approach would be to increase the identification of viral sequences that may impact human health. Indeed, in virome studies, most of recovered contigs belong to phages, or animal viruses making human virus sequence identification difficult as some animal viruses are closely related to Human strains (Guajardo-Leiva et al., 2020; Martinez-Puchol et al., 2020; McCall et al., 2020). Human enteric viruses usually have single-strand RNA genomes, relatively short (around 7 kb), some being segmented as for rotavirus, making precise identification complex. Moreover, these RNA molecules are fragile and may be destroyed during sample preparation and the different purification steps. From a public health perspective, obtaining long contigs allowing viral genomes identification and investigation of transmission chains constitute a gold standard but remains a challenge when dealing with complex environmental samples such as wastewater (Aarestrup et al., 2021; Cobbin et al., 2021; Mazur et al., 2022). In a recent comment, Diamond et al. (2022) highlight the need to improve sewage sample analysis to be able to distinguish signal from noise to rapidly identify emerging Human pathogen (Diamond et al., 2022).

To achieve our goal to obtain long contigs allowing characterization of viral genomes, we used a previously described method (Bisseux et al., 2018; Strubbia et al., 2019). Combining a classic approach such as PEG precipitation, and additional purification steps including a special care given to protect viral RNA up to the guanidinium thiocyanate solution treatment contribute to successfully identify viral sequences.

Another issue when analyzing samples such as sewage is the representativeness of the sampling. Collecting 24-h composite sample increase the probability to detect a large diversity of strains compare to a grab sample, but the issue of low concentration of some viruses compared to other micro-organisms remains (Corpuz et al., 2020; Ahmed et al., 2022). One option can be to concentrate a large volume of wastewater, but this increases the risk to concentrate inhibitors of enzymes used during the library preparation (McCall et al., 2020; Adriaenssens et al., 2021). Instead, we chose to perform biological replicates, that is to repeat several extractions from the same sample and on different series (performed on different days) to limit any bias between samples. To evaluate the impact of the multiple purification steps in our method, and to verify that replicates provide comparable results, a control step in the form of a norovirus-specific qPCR was performed on the cDNA to verify that this viral target was not lost during the sample preparation up to RT. Overall, all replicates but one provided comparable results for norovirus GII to initial analysis, while for norovirus GI, more variations were observed presumably linked to the lower concentration of this genogroup as usually observed in sewage (Miura et al., 2018; Fumian et al., 2019). Importantly, this verification showed concentrations of norovirus (GI + GII) similar or somewhat higher than the initial one-step RT-qPCR screening, suggesting an efficient recovery of viral particles despite the multiple steps of the protocol, and a better elimination of inhibitors, compared to the initial screening approach. This norovirus quantification was used as a proxy to estimate the efficacy of the process to obtain viral genomes, assuming that other human enteric viruses behaved similarly. Adding some controls at different steps of sample preparation seems to be an important issue for further developments and subsequent risk assessment (Diamond et al., 2022; Fu et al., 2022).

In addition to the care given to sample preparation, we used a target enrichment step based on probe capture, whose added-value in yielding sequences of interest has been demonstrated in clinical, sewage or shellfish samples (Briese et al., 2015; Strubbia et al., 2019; Martinez-Puchol et al., 2020; Bonny et al., 2021). This enrichment step during the library preparation was shown efficient to identify novel viral sequence even in complex samples, however the relative abundance of the different sequences may be modified (Briese et al., 2015). Also, as we expected to recover a low proportion of viral reads, we chose a high sequencing depth by performing a Next-seq (Guerrero-Latorre et al., 2018; McCall et al., 2020; Nieuwenhuijse et al., 2020). Indeed, combining this enrichment approach and high numbers of reads allowed to eliminate a large percentage of no-viral reads while still having enough reads to identify. When processing the reads into contigs it is important to have a good coverage to be confident in the contigs identification. In our study, the merged replicates datasets before *de novo* assembly yielded longer viral contigs with better coverage. Such approach allowed us to set up quality criteria on read numbers contributing to the contigs and on contig length of at least a thousand bases for viral identification. However, this was not always sufficient to precisely identify some sequences for highly diverse viruses such as enterovirus or norovirus. Indeed, for these viruses, some of the obtained sequences just allowed genus identification, because they fell outside the genomic regions used for genotyping. Considering the large richness of micro-organisms present in wastewater samples, to further investigate any public-health impact or risk assessment, some authors suggest to add some additional testing such as specific RT-PCR or amplicon deep sequencing (Martinez-Puchol et al., 2020; Adriaenssens et al., 2021; Yang et al., 2021; Fu et al., 2022).

Our approach combining a deep sequencing, merged analysis of technical replicates, and the capture enrichment step allowed to identify several complete genomes of viral families that infect humans. For example, up to 12 complete sequences of mamastrovirus were identified. *Astroviridae* family sequences are frequently detected in sewage samples and a large diversity of strains may be present (Rodriguez-Diaz et al., 2009; Hata et al., 2018; Yang et al., 2021). The other complete genomes were mainly norovirus (four complete GII.6[P7]), one Hepatitis E virus genotype 3f and one Hepatitis A virus (genotype 1A). As mentioned above, samples selected for this study were collected in different years, months and locations, to lower bias due to sites or wastewater compositions related to one specific site. These samples were included based on previous data from the two samples collected in a small island, where two hepatitis outbreaks occurred: one due to hepatitis E virus following a piglet consumption in 2013 and one due to hepatitis A virus linked to a person working in a restaurant (Guillois et al., 2016; and unpublished data). Unfortunately, no wastewater sample collected during the 2013 hepatitis E outbreak was available. At this time, we were able to characterize a small portion of the genome from one positive sewage sample, close to the sequence identified in patients and in the pig farm (Guillois et al., 2016; Miura et al., 2016). Interestingly an almost complete genome of the same subtype 3f was identified in the sample collected in site K in 2014, few months after this hepatitis E outbreak (Figure 7). While no hepatitis E virus read was identified in the sample collected in 2012, two other identical sequences similar to this 2014- sequence were identified in samples collected in this WWTP K two year later. This WWTP collects sewage from a small city close to the island (Figure 1), suggesting that some secondary cases occurred in the city, some probably being asymptomatic as observed during the epidemiological investigation of the initial outbreak (Guillois et al., 2016). In 2016, following the hepatitis A outbreak, some sewage samples collected from the small island were positive for hepatitis A virus by RT-PCR (unpublished data). Here, we were able to obtain a full genome sequence of a genotype IA, genotype circulating in France (Bisseux et al., 2018). These results confirmed the importance of metagenomic to identify viral sequences and contribution that can be made to epidemiological surveillance (Izopet et al., 2019; Diamond et al., 2022; Munoz-Chimeno et al., 2022). This also reinforce the need to associate environmental surveillance with epidemiological data.

As mentioned above despite the care given to purify viral genomes and to increase the number of viral reads the issue of contig identification remains critical for segmented genomes. Database such as NCBI Genbank or more dedicated tool like the norovirus typing tool or the Rotac tool (for rotavirus) are really helpful. However, assembling the 11 rotavirus genes from a sewage sample, remain a delicate challenge, performed on previously described constellations obtained in Human or animal stool samples (Simsek et al., 2021; Portal et al., 2022). It is interesting to note that some genes were more frequently identified in our samples than other without any relationship to the length. This issue may need to be further investigated before wastewater samples can be used to evaluate the genetic diversity of the different segments and any changes in the patterns of the genotype distribution (Matthijnssens and Van Ranst, 2012). We also faced this issue for the analysis of the bi-segmented double-stranded RNA genomes of the picobirnavirus. Many complete segments could not be clearly identified in GenBank, but the high number of contigs detected in this study suggest that picobirnaviruses may be under-detected in the human population, as hypothesized also by others (Ghosh and Malik, 2021).

The aim of this study was to evaluate the relevance of technical replicates and quality criteria such as a minimum of reads or contigs length to identify human pathogenic virus sequence with confidence. We chose samples from diverse WWTP and different seasons to ensure that our protocols could be applied to diverse wastewater samples. As some samples were kept frozen for more than 10 years and could be degraded, we did not expect to raise conclusion in terms of sequence diversity or the presence of specific strains. Yet, we included samples linked to hepatitis outbreaks to assess the possibility to detect the causing agents retrospectively. Our approach was found efficient to identify some sequences and successfully described the viral diversity, yielding full genomes either for norovirus, enterovirus or even rotavirus, even if, for these segmented genomes, combining genes remains a difficult issue.

Metagenomics allowing the description of all genomes present in a sample applied to wastewater samples that received all different viruses replicating in the gut of the local population constitute a promising approach for epidemiological surveillance. Our results show that performing biological replicates and merging the obtained data allow to obtain longer contigs and a reliable strain identification, an important issue for public health surveillance. Indeed, confidence in result interpretation is strengthened by quality criteria in terms of read numbers and contig length (Diamond et al., 2022). Importantly, this wastewater-based approach yielded viral sequences, sometimes whole genomes, that could be phylogenetically related to those obtained during related outbreak investigations or public databases. With the improvements of methods and the enrichment of databases, wastewater sample analysis will become an important tool also to prevent further transmission by raising alerts in case of viral outbreaks or emergence.

## Data availability statement

The sequencing data presented in this study are deposited in the NCBI repository. Accession numbers can be found in Supplementary Table 1.

## Author contributions

JS, MD, and FG contributed to the conception, design of the study, contributed to data interpretation, and manuscript preparation. JS performed experiments. JS and AB performed the bio-informatics analysis. All authors contributed to the article and approved the submitted version.

## Funding

This study was supported by the COMPARE and VEO European projects (H2020 grant agreement N°643476 and H2020 SC1-2019-874735) and by support from the Direction Générale de l’Alimentation (DGAl) through the annual convention.

## Conflict of interest

The authors declare that the research was conducted in the absence of any commercial or financial relationships that could be construed as a potential conflict of interest.

## Publisher’s note

All claims expressed in this article are solely those of the authors and do not necessarily represent those of their affiliated organizations, or those of the publisher, the editors and the reviewers. Any product that may be evaluated in this article, or claim that may be made by its manufacturer, is not guaranteed or endorsed by the publisher.
